# Exploring the Use of Telemonitoring for Patients at High Risk for Hypertensive Disorders of Pregnancy in the Antepartum and Postpartum Periods: Scoping Review

**DOI:** 10.2196/15095

**Published:** 2020-04-17

**Authors:** Maria Aquino, Sarah Munce, Janessa Griffith, Maureen Pakosh, Mikayla Munnery, Emily Seto

**Affiliations:** 1 Institute of Health Policy, Management and Evaluation Dalla Lana School of Public Health University of Toronto Toronto, ON Canada; 2 Centre for Global eHealth Innovation Techna Institute University Health Network Toronto, ON Canada; 3 Rumsey Centre Toronto Rehabilitation Institute University Health Network Toronto, ON Canada; 4 Institute of Medical Science University of Toronto Toronto, ON Canada; 5 Women’s College Hospital Institute for Health System Solutions and Virtual Care Women’s College Hospital Toronto, ON Canada; 6 Library & Information Services Toronto Rehabilitation Institute University Health Network Toronto, ON Canada

**Keywords:** high-risk pregnancy, blood pressure, preeclampsia, telemetry, telemedicine, mHealth, eHealth, smartphone, mobile phone

## Abstract

**Background:**

High blood pressure complicates 2% to 8% of pregnancies, and its complications are present in the antepartum and postpartum periods. Blood pressure during and after pregnancy is routinely monitored during clinic visits. Some guidelines recommend using home blood pressure measurements for the management and treatment of hypertension, with increased frequency of monitoring for high-risk pregnancies. Blood pressure self-monitoring may have a role in identifying those in this high-risk group. Therefore, this high-risk pregnancy group may be well suited for telemonitoring interventions.

**Objective:**

The aim of this study was to explore the use of telemonitoring in patients at high risk for hypertensive disorders of pregnancy (HDP) during the antepartum and postpartum periods. This paper aims to answer the following question: What is the current knowledge base related to the use of telemonitoring interventions for the management of patients at high risk for HDP?

**Methods:**

A literature review following the methodological framework described by Arksey et al and Levac et al was conducted to analyze studies describing the telemonitoring of patients at high risk for HDP. A qualitative study, observational studies, and randomized controlled trials were included in this scoping review.

**Results:**

Of the 3904 articles initially identified, 20 met the inclusion criteria. Most of the studies (13/20, 65%) were published between 2017 and 2018. In total, there were 16 unique interventions described in the 20 articles, all of which provide clinical decision support and 12 of which are also used to facilitate the self-management of HDP. Each intervention’s design and process of implementation varied. Overall, telemonitoring interventions for the management of HDP were found to be feasible and convenient, and they were used to facilitate access to health services. Two unique studies reported significant findings for the telemonitoring group, namely, spontaneous deliveries were more likely, and one study, reported in two papers, described inductions as being less likely to occur compared with the control group. However, the small study sample sizes, nonrandomized groups, and short study durations limit the findings from the included articles.

**Conclusions:**

Although current evidence suggests that telemonitoring could provide benefits for managing patients at high risk for HDP, more research is needed to prove its safety and effectiveness. This review proposes four recommendations for future research: (1) the implementation of large prospective studies to establish the safety and effectiveness of telemonitoring interventions; (2) additional research to determine the context-specific requirements and patient suitability to enhance accessibility to healthcare services for remote regions and underserved populations; (3) the inclusion of privacy and security considerations for telemonitoring interventions to better comply with healthcare information regulations and guidelines; and (4) the implementation of studies to better understand the effective components of telemonitoring interventions.

## Introduction

### Background

High blood pressure is one of the most common complications during pregnancy, affecting 2% to 8% of pregnancies [1,2]. The spectrum of hypertensive disorders of pregnancy (HDP) includes chronic hypertension, gestational hypertension, preeclampsia, eclampsia, and preeclampsia superimposed on chronic hypertension [1]. HDP pose short- and long-term risks for maternal and fetal health, including preterm delivery; hemolysis, elevated liver enzymes, and low platelet (HELLP) syndrome; disseminated intravascular coagulation; chronic renal failure; coronary artery disease; and premature death [3-5]. In addition, eclampsia and HELLP syndrome can occur for some patients postpartum [6-9]. Eclampsia, which is the new onset of grand mal seizures occurring in the absence of another identifiable cause [10], can develop at any point antepartum (38%-53%), intrapartum (18%-36%), and postpartum (11%-44%) [7]. Signs of preeclampsia usually diminish by 6 to 12 weeks postpartum [10].

During pregnancy, blood pressure measurements are routinely monitored, and high blood pressure is identified using blood pressure values obtained at the hospital or clinic visit [11]. International guidelines, such as the American College of Obstetricians and Gynecologists Hypertension in Pregnancy Guidelines for the treatment of hypertension, define it as clinic blood pressure >140/90 mm Hg [11]. Compared with clinic blood pressure measurements, ambulatory blood pressure measurements (ie, blood pressure measurements collected at various points, usually in a 24-hour period, while patients conduct routine activities) and home blood pressure measurements (ie, blood pressure values collected under a fixed schedule over a prolonged timeframe) [12] have a stronger association with end-organ damage and long-term health effects in the general population outside of pregnancy [13-15]. In fact, some international guidelines, such as the American Heart Association, American Society of Hypertension, Preventive Cardiovascular Nurses Association joint statement [16], and the European Society of Hypertension guidelines, emphasize the importance of blood pressure self-monitoring [17].

The benefits of home blood pressure monitoring have been demonstrated for clinical decision making. For example, home blood pressure between clinic visits can help to identify blood pressure changes in pregnant patients at home [17] and, combined with demographic risk factors such as chronic kidney disease, diabetes mellitus, and autoimmune disease such as systemic lupus erythematosus [18] may allow clinicians to estimate a woman’s risk for HDP. Current UK guidelines recommend increased blood pressure monitoring for those at higher risk of preeclampsia [18]. Self-monitored blood pressure readings may have a role in identifying those in this high-risk group as well as those with white-coat hypertension (ie, high clinic blood pressure measurements but normal blood pressure otherwise) [19] and true chronic or gestational hypertension [20]. In addition, the 2013 guidelines from the American College of Obstetricians and Gynecologists recommend home monitoring for pregnant patients with chronic, poorly controlled, and gestational hypertension [21], and the National Institute for Health and Care Excellence recommends the self-monitoring of preeclampsia symptoms [22]. In general, the World Health Organization (WHO) recommends pregnant women to maintain their own case notes or home-based records related to their pregnancy to improve continuity and quality of care [23].

Telemonitoring may be well suited for patients at high risk for HDP for several reasons. First, more pregnant patients are tracking home blood pressure measurements through their own volition or by instruction from their health care provider (HCP) [24]. Second, pregnant patients who require additional monitoring have indicated a preference for the self-measurement of blood pressure at home over the more frequent visits to the prenatal clinic or ambulatory blood pressure monitoring [25,26]. Furthermore, telemonitoring has been shown to be effective for the management of chronic conditions, including cardiopulmonary disease, asthma, and heart failure, which have contributed to reduced patient travel, absenteeism, hospital length of stay, readmissions [27], and overall costs [28].

### Objectives

There are three published reviews related to the telemonitoring of pregnant patients using various technologies. In 2017, Lanssens et al [29] published a scoping review on the telemonitoring of patients during the prenatal period. However, their review primarily focused on the telemonitoring of patients at high risk for preterm labor and gestational diabetes [29]. In addition, Lanssens et al [29] employed a narrow time range, from 1988 to 2010, for their inclusion criteria, potentially leading to missed relevant publications before 1988 and after 2010. Few studies have explored the existing telemonitoring technologies during the prenatal period, and in fact, the review by Lanssens et al [29] identified only 14 papers on the effectiveness of telemonitoring in obstetrics. Rivera-Romero et al [30] published a scoping review in 2018, which identified 11 articles exploring mobile health (mHealth) solutions for HDP. The authors found that only four studies collected physiological data and only two studies collected blood pressure measurements. Van den Heuvel et al [31] described 71 studies that reported on electronic health (eHealth) use during prenatal, perinatal, and postnatal care. In their review, the authors found 12 studies describing telemonitoring and teleconsulting interventions and stated that telemonitoring of pregnancy may be a mechanism through which the potential of eHealth technologies is realized [31]. This scoping review aims to describe the available studies on telemonitoring interventions for the detection and management of HDP. This paper aims to answer the following research question: What is known about telemonitoring interventions for the management of patients at high risk for HDP? Specifically, this study sought to understand the types of telemonitoring interventions that have been used, the study designs employed, and the results of the studies.

## Methods

### Literature Review

A literature review consistent with the Preferred Reporting Items for Systematic Reviews and Meta-Analyses Extension for Scoping Reviews (PRISMA-ScR) guidelines and following the methodological framework described by Arksey et al [32] and Levac et al [33] was conducted to analyze studies describing the telemonitoring of adult patients at high risk for HDP.

### Inclusion Criteria

Peer-reviewed studies were included if they met the following criteria: (1) published in English, (2) included antepartum and postpartum adults aged 18 years and older at high risk for HDP (ie, history of high blood pressure, advanced maternal age, elevated blood lipids, high body mass index, and history of diabetes mellitus) [34], and (3) described a telemonitoring intervention that included feedback from the HCPs to the patients. For this scoping review, telemonitoring interventions were defined in the same way as described by Lanssens et al [29], involving periodic measurements of physiological metrics (eg, blood pressure, weight, and physical activity) and using an information and communication technology to relay these metrics from the patient’s home to a health care facility. These telemonitoring interventions included measurements taken by patients themselves as well as through home visits by HCPs or community workers to ensure a comprehensive understanding of the available models of telemonitoring. All study designs were included in this review, such as randomized controlled trials (RCTs), prospective and retrospective studies, feasibility studies, economic evaluations, and case studies. No limitations with respect to year of publication were imposed. Abstracts, books and book chapters, literature reviews, and research in progress were excluded because the intent was to review completed research and peer-reviewed publications of telemonitoring interventions for patients at high risk for HDP.

### Search Strategy

A comprehensive literature search was conducted in July 2018 in the following databases: Medical Literature Analysis and Retrieval System Online (MEDLINE), PubMed, Cochrane Database of Systematic Reviews, Cochrane Central Register of Controlled Trials, Cumulative Index Nursing and Allied Health Literature, PsycINFO, Excerpta Medica Database, and EMCare.

Search strategies were developed with the aid of an experienced information specialist (MP) who reviewed and refined them through discussion with the research team. Keywords for this review included the target population, health condition, and telemonitoring system (ie, telemonitoring of adult patients at high risk for HDP). An article from a manual review of journals was identified and added to the search results for screening. The final search strategy for MEDLINE can be found in Multimedia Appendix 1.

### Selection Procedure

**Figure 1:**
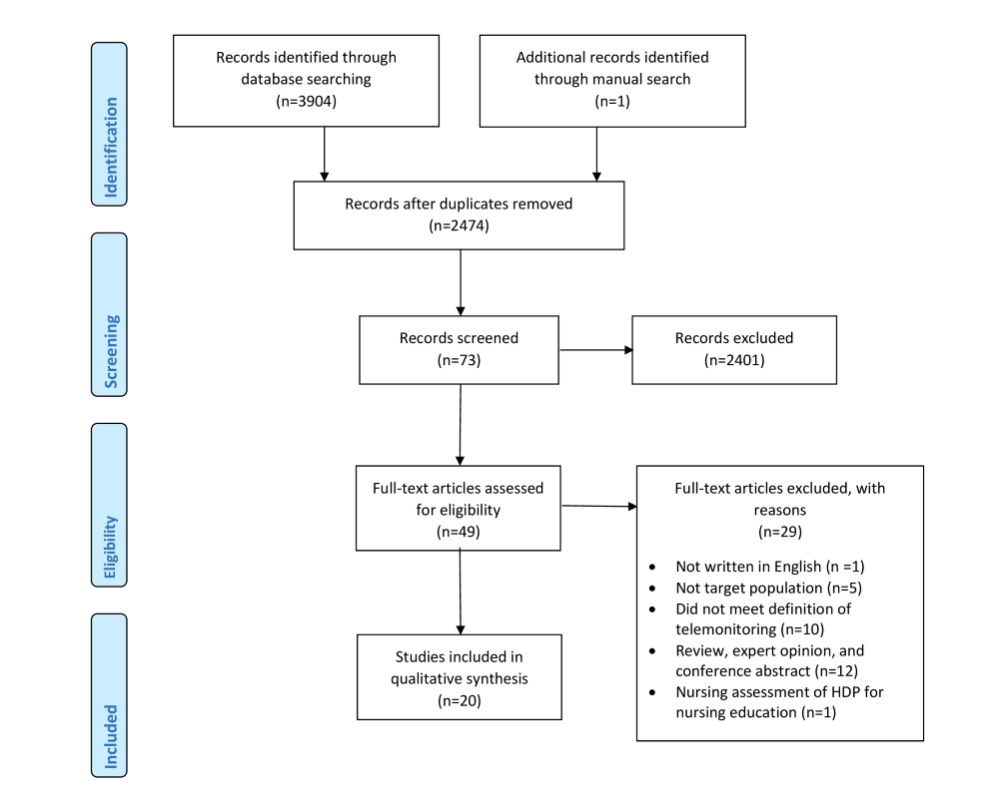
Preferred Reporting Items for Systematic Reviews and Meta-Analyses flow diagram. HDP: hypertensive disorders for pregnancy.

Selection criteria forms were developed by the primary reviewer (MA) and pilot tested with the research team using five randomly selected articles. This was done to facilitate consistency among reviewers in the selection process. The initial database search resulted in 3904 articles. The selection procedure is illustrated in the flow diagram in Figure 1. After duplicate articles were removed, 2474 articles remained. The titles and abstracts of 2474 publications were independently evaluated and assessed for eligibility by two sets of reviewers (complete review by MA and joint review by SM and JG) using the systematic reviews web app Rayyan Qatar Computing Research Institute (Hamad Bin Khalifa University, Doha, Qatar) [35]. Of these, 49 publications were found to be eligible by 2 reviewers. The full text of the 49 publications were reviewed independently by MA and JG. A total of 29 publications were excluded because of the following reasons: (1) the article was not written in English; (2) the study did not include our target population; (3) the study did not meet our definition of telemonitoring; (4) the article was a review, conference abstract, or featured expert opinion; or (5) the study focused on the nursing assessment of HDP for nursing education. Disagreements among the reviewers were resolved by discussion until consensus was reached. A total of 20 articles were included for full data extraction and analysis.

### Data Extraction

A data-charting table was developed collectively with the research team to determine relevant variables to include, and it was pilot tested to facilitate consistency during data extraction. A total of two sets of reviewers (complete review by MA and joint review by MM and JG) independently completed data extraction from each of the 20 articles. Any disagreements among the reviewers were resolved through discussion. Relevant data from the included studies were recorded on the data-charting table, including information on study characteristics, telemonitoring intervention description, outcome measures, and results.

## Results

### Study Characteristics

The included papers were published from 1987 to 2018, with more than half (n=13) published in 2017 (n=5) and 2018 (n=8). A total of eight [36-43] papers were published in the United Kingdom, followed by the United States (n=4) [44-47], Belgium (n=4) [48-51], Dominican Republic (n=1) [52], Netherlands (n=1) [53], and Guatemala (n=1) [54]. One study [55] described two different versions of a telemonitoring intervention being implemented in Nigeria, Mozambique, Pakistan, and India. A total of 15 papers reported on antepartum women, whereas five papers reported on postpartum women (refer to Multimedia Appendices 2 and 3 for further details).

A total of three study designs were identified in the included articles: a qualitative study (n=1), observational studies (n=16), and RCTs (n=3). A total of seven articles reported on the distinct aspects of the same three studies: for the blood pressure self-monitoring in pregnancy study, an observational study [37] and a qualitative study [38] were conducted; for the pregnancy remote monitoring study, observational studies at 1- [48] and 2-year [49] intervals as well as a cost-benefit analysis [50] were conducted; and for the home blood pressure monitoring study, two observational studies [36,39] were conducted. Thus, there were a total of 16 unique interventions from the 20 included studies. To avoid duplication, papers reporting on similar study results are grouped together.

The total number of study participants for the included studies was 2709, with one study [55] predicting the telemonitoring of over 30,000 women when its interventions are implemented. Study participants from two studies [39,48] were disregarded in the final tally because study participants in these studies were already accounted for in another study. There were no standardized criteria for the suitability of patients undergoing blood pressure telemonitoring.

### Critical Appraisal of Included Studies

In keeping with the PRISMA-ScR guidelines, an assessment of the methodological approaches of the included articles was performed to identify study limitations. Included studies were assessed by study design (ie, qualitative studies, observational studies, and RCTs), and emerging themes were extracted from each grouping of studies.

The key themes that emerged from the qualitative and observational studies were as follows: (1) small study sample sizes; (2) nonrandomized study groups; (3) demographic and characteristic differences among groups; (4) short study durations; (5) limitations of study findings to the clinical and regulatory practices of a particular country; and (6) costs analyses that did not account for other costs to the patient, such as transportation, travel costs, and lost income for time spent in the hospital or clinic visit. For the three RCTs, investigators were unable to blind the study personnel and participants to the intervention, and there was a lack of allocation sequence concealment.

### Intervention Characteristics

The study duration for interventions during pregnancy ranged from a gestational age of 4 weeks to delivery or admission to the hospital, whereas interventions in the postpartum period ranged from after delivery to 6 months. A total of three studies [43,51,55] did not specify the duration of their interventions. The data collection period of the studies ranged from 7 months to 35 months for interventions during pregnancy, whereas the data collection period for studies in the postpartum period ranged from 4 to 12 months.

All interventions collected maternal blood pressure, with some interventions collecting additional metrics such as heart rate; weight; activities; urinalysis for glucose and protein; symptoms of preeclampsia such as headache, epigastric pain, and visual symptoms; temperature; peripheral capillary oxygen saturation; and psychosocial signs and symptoms. In addition, four articles [51-54] collected data from the fetus, including fetal heart rate and kick count. A total of six unique studies [37,38,41-43,45,46] reported interventions in which only maternal blood pressure was collected. Blood pressure thresholds varied for each intervention, with alarm thresholds ranging from >140/90 mm Hg (n=5) to >160/110 mm Hg (n=4), or were unspecified (n=7). Criteria for interventions with blood pressure thresholds were either applied using guidelines, such as the International Society for the study of Hypertension in Pregnancy [36,48-50], American College of Obstetricians and Gynecologists [45,46], and local clinic guidelines [37,38,42], or were unspecified [41,43].

The schedule for collecting maternal blood pressure measurements varied across the included studies and ranged from four times a day (n=1) [47], two times a day (n=8) [37,38,44-46,48-50], daily (n=2) [40,53], weekly (n=1) [43], to did not specify a schedule (n=4) [42,51,54,55]. A total of three unique studies [36,39,41,52] proposed a variable schedule depending on patient condition.

### Intervention Design and Implementation

All 16 interventions provided clinical decision support. A total of four of these interventions were developed specifically to enhance the assessment capabilities of HCPs, including Indigenous Mayan traditional birth attendants [54], community-based HCPs [55], community health workers [52], and physicians and midwives [53]. The remaining 12 interventions also facilitated the self-management of pregnant patients’ signs and symptoms related to HDP.

Instructions in response to abnormal signs and symptoms were provided to patients for self-management interventions manually (n=6) [44,47-51], automatically (n=4) [40,43,45,46], or both (n=4) [36,38,39,56], or they were unspecified (n=2) [42,57]. Manual instructions for patients and HCPs included written or verbal instructions, and automatically provided instructions were given through a digital platform, such as a mobile or web app or a web-based dashboard.

Physiological data from interventions developed for enhancing HCP assessment capabilities (n=4) were inputted by HCPs when they visited patients at home, whereas interventions with the additional self-management component (n=12) were inputted by the patient. Physiological data of pregnant patients were entered either manually or automatically. Manual input included writing results of the patients in a journal [36-39,42], texting [45,46], or calling [47] to convey these results to HCPs. Automatic input involved a real-time transfer of blood pressure readings from an automated blood pressure machine to a digital platform. Automatic input included the use of a mobile phone or smartphone [36,38-40,43,52,54,55], whereas other technological components involved a web-based dashboard [48-50], web app [46,51], modem [41,53], or cloud-based portal [44].

The clinical team of HCPs, such as nurses, physicians, obstetricians, midwives, traditional birth attendants, community health workers, and community HCPs, who managed and supported pregnant patients at home varied depending on the intervention. A total of three studies [44,45,47] described interventions with 1 designated HCP who reviewed and monitored pregnant patients, whereas most studies (n=17) defined a more team-based approach in which a nurse or midwife triaged patients and consulted with an obstetrician on recommendations for the patient.

A total of 11 studies [36-39,41,42,44-47,51] described a training session for patients on how to use the intervention and provided information for the normal and abnormal values of physiological metrics, as well as actionable steps for critical results. A total of six studies [40,43,48-50,53] did not specify a training plan for patients or HCPs, and three studies [52,54,55] described training for HCPs. Training for HCPs to use interventions to enhance their assessment capabilities tended to be more intensive than training for patients to facilitate the self-management of HDP. For example, training for traditional birth attendants in Guatemala involved a 4-day training session by nurses on key maternal and neonatal assessment concepts, a minimum passing score of 90% for three standardized patient encounters, and retraining and reevaluation sessions for those who failed the initial evaluation in addition to training on intervention use and knowledge of normal and abnormal values [54]. Similarly, training for community health workers in San Juan Province, Dominican Republic, entailed a 2-day workshop, an overview of maternal anatomy and obstetric assessment, 8 days of individual training, ongoing evaluations through discussion, observation, and a return demonstration [52].

The use of theoretical frameworks to guide the intervention was uncommon, with only one study describing the use of a theoretical framework in its implementation of an intervention. Bonnell et al [52] employed a community-based participatory research approach in examining how mHealth technology can be used in a community health worker model in San Juan Province, Dominican Republic.

### Intervention Technology and Home Monitoring Platform

As all interventions collected maternal blood pressure readings, all interventions included the use of blood pressure monitors. A total of 11 interventions, described in 15 studies [36-41,43-51], provided blood pressure monitors to patients, whereas the other four interventions [52-55] required HCPs to take the patient’s blood pressure readings during home visits. One intervention [42] did not specify whether the blood pressure monitor was provided to the patient for home use or whether HCPs brought along the monitors during home visits. Interestingly, not all interventions using blood pressure monitors were validated for pregnancy and preeclampsia. In fact, only three interventions, described in five studies [36-40], used the Microlife WatchBP monitor, which is validated for use in pregnancy and preeclampsia [58]. One intervention [54] used the Omron M7 monitor, which is validated for pregnancy but not for severe preeclampsia [59]. A total of two unique interventions [45,48-50] used blood pressure monitors that were not validated for use in pregnancy or preeclampsia (ie, Withings Blood Pressure Monitor) [60,61] or required consultation with a physician before use in pregnant patients (ie, Omron 3 series Blood Pressure Monitor) [62]. A total of eight interventions [41-43,46,51-53,55] did not specify the blood pressure monitor or model used, and validation information on 2 blood pressure monitors (eg, Ideal Life and Vasoplex) [44,47] could not be found. Additional technological components for each intervention depended on supplementary physiological metrics that were collected, such as a pulse oximeter, urine dipsticks, fetal heart monitor, activity tracker (ie, Withings Smart Body Analyzer), thermometer, and scale.

### Study Results and Outcomes

Included studies reported on four main outcomes: (1) maternal and fetal outcomes, (2) health system utilization, (3) user (ie, patient or HCP) experience, and (4) intervention feasibility (see Multimedia Appendix 4 for further details).

A total of eight studies [36,39,40,44,48,49,53,54] reported on maternal and fetal health outcomes. Maternal outcomes included medication adherence, gestational outcomes, mode of delivery (eg, cesarean or vaginal), and adverse outcomes (including but not limited to acute renal failure, acute myocardial ischemia, intravenous medication for blood pressure control, hypertensive encephalopathy, and death). Fetal outcomes included neonatal and adverse outcomes such as preterm delivery, small for gestational age, fetal growth restriction, and death. A total of two unique studies [48,49,53] reported significant findings for the telemonitoring group, namely, spontaneous deliveries were more likely to take place. One study, described in two papers, reported that inductions were less likely to take place for patients in the telemonitoring group compared with the control group (*P*<.01) [48,49]. A total of six studies [36,39,40,44,53,54], which included two RCTs [40,53], reported nonsignificant findings regarding medication adherence and maternal or fetal health outcomes for patients in the telemonitoring group.

Health system utilization outcomes included admission and readmission or referral to the hospital, admission to the neonatal intensive care unit, and costs associated with admission to the hospital. A total of 11 papers reported on health system utilization. Moreover, two studies described more return visits to a health care facility: one study noted that mHealth users returned to a medical facility compared with none of the nonusers (*P*=.004) [44]. Another study reported that women using a mobile app to monitor home blood pressure returned to the hypertension clinic more times than the nonapp-based home blood pressure monitoring and control groups (*P*<.001) [39]. However, the authors did not explain whether an escalation of care was required and whether the cause of the return visit was related to hypertension. A total of four studies [36,39,48,49] reported fewer prenatal hospitalizations and clinic visits for patients in the telemonitoring group compared with the conventional monitoring group. Another study [53] reported no difference between the groups with regard to maternal and neonatal hospital admission rates. Furthermore, three studies [39,50,51] described cost savings as a result of fewer hospitalizations and clinic visits. One study [45] reported no hospital readmission in a postpartum population undergoing telemonitoring. Martinez et al [54] described an increase in referral rates to facility-level care when traditional birth attendants had access to the mHealth intervention.

Overall, five studies [38,43,45,47,52] reported on user (eg, patient or clinician) experience, which included the intervention’s ease of use, and users’ preference for and perceptions of blood pressure self-monitoring at home. A total of four of the five studies [38,43,45,47] discussed patients’ preference for and perceptions of telemonitoring interventions compared with hospital or clinic visits. In addition, patients with a previous history of preeclampsia perceived that having the telemonitoring intervention empowered and reassured them [38]. Bonnell et al [52] reported that patients felt that home visits by community health workers using an mHealth app for the monitoring and assessment of HDP were of comparable clinical value to prenatal visits to local health care facilities.

Intervention feasibility included perceived benefits and barriers, data accuracy, recruitment, compliance, and effectiveness of detecting HDP. In one study, patients who elected to be in the telemonitoring group perceived telemonitoring to be beneficial [44]. Other studies found that home blood pressure readings were accurately collected or transferred to a digital platform [40,42,43] and that HCPs were able to reliably use home blood pressure measurements for clinical decision making in a similar way as clinic blood pressure measurements [37,47,54]. In addition, three interventions described a high rate of compliance related to blood pressure reading collection and submission. One study found that 84% of the participants texted at least one blood pressure measurement [45], whereas another study found that 92.2% of the participants from the telemonitoring group sent one blood pressure measurement via text message compared with the 43.7% of the participants from the control group who sent one blood pressure measurement via text message [46]. The median compliance was 85% for submitting daily blood pressure readings for another study [40]. However, the lack of consistent internet connection in rural areas proved to be a technological barrier to telemonitoring [52].

## Discussion

### Principal Findings

This review presented a summary of existing telemonitoring interventions for patients at high risk for HDP both antepartum and postpartum. Of the 20 included studies, 13 were published between 2017 and 2018, suggesting that telemonitoring interventions for patients at high risk for HDP are a novel and burgeoning area of research. This study, which only identified 16 unique interventions, reflects the limited use of telemonitoring for antepartum [29] and postpartum [44] patients. Interventions for postpartum patients were included because current recommendations call for the postpartum monitoring of hypertension, as symptoms can develop regardless of the history of hypertension or preeclampsia in the antepartum period [6]. However, given the exploratory nature of a portion of the included studies, more research is needed before any recommendations can be made for the telemonitoring of this high-risk group.

Surprisingly, not all interventions used blood pressure measurement monitors that are validated for use in pregnancy and preeclampsia. Only five studies reported using a blood pressure instrument validated for pregnancy and preeclampsia, such as the Microlife WatchBP monitor. Accurate blood pressure measurements in pregnancy are essential for the appropriate management and treatment of patients [63]. HCPs should be cognizant of the impact of inaccurate blood pressure measurements and should consider recommending patients to use blood pressure measurement monitors that have been appropriately validated in this population [63]. Finally, implementing the practice of blood pressure self-monitoring in the antepartum and postpartum period would require a standardization of home blood pressure thresholds [20].

The feasibility [40,42,43] and accuracy [37,47,54] of blood pressure readings from the included interventions support the WHO recommendation of pregnant women maintaining home-based records throughout their pregnancy [23]. There was also a high compliance rate noted by three interventions, but only one of these interventions discussed security as an intervention consideration [45]. In patients with gestational diabetes mellitus, Homko et al [64] showed a significant correlation between income and blood sugar result transmissions, with women with higher income sending results more frequently. Therefore, consideration of socioeconomic status may be beneficial when developing and implementing self-management interventions. Given the ubiquitous nature of mobile phone use [65], there is potential for integrating mobile phones and smartphones into the management and treatment of this patient population. In addition, automatic data entry of blood pressure measurements may reduce the possibility of incorrectly entering blood pressure measurements and the burden associated with manual entry.

This review showed only two unique studies reporting a significant difference for maternal and fetal health outcomes, with the telemonitoring group experiencing a higher likelihood for spontaneous deliveries [48,49,53], and one study reported a lower likelihood for inductions [48,49]. Similarly, previous studies reporting on the health outcomes of women with gestational diabetes using mHealth technologies showed limited impact on maternal health outcomes [64,66,67]. Homko et al [64] concluded that the benefits of health care technologies may lie in their ability to streamline health care processes (eg, reducing the need for clinic follow-ups and unnecessary hospital admissions). Costs analyses included in this review highlight the ability for telemonitoring to reduce costs by monitoring stable high-risk pregnant women at home [39,50,51] instead of being admitted to a hospital, which is the standard management for patients diagnosed with preeclampsia [68,69]. As outpatient management of HDP occurs in the earlier stages of pregnancy [24] and after the baby has been delivered [68], telemonitoring may provide enhanced surveillance of disease progression. However, telemonitoring interventions should not encourage obstetricians to defer hospital admission [24] or replace HCP visits or contact points. The WHO recommends a minimum of eight contact points with an HCP to decrease antenatal mortality and improve women’s experience during pregnancy [23]. Contraindications to the self-monitoring of home blood pressure, such as atrial fibrillation or other abnormal heart rhythms [16], should also be considered.

Identified barriers in the implementation of telemonitoring interventions for this high-risk group include mHealth infrastructure [52,55] and costs (eg, equipment or lack of HCP compensation for the provision of telemedicine) [44]. Similarly, a systematic review on the use of mHealth technologies for the self-management of diabetes noted the following barriers for its adoption and use: a lack of financial resources, constrained human and technical resources, integration challenges with existing health information systems, and limited incentives or reimbursement [70].

Despite the limitations of telemonitoring, the included studies generally showed telemonitoring to be positive for patients and HCPs. Patients stated their preference for telemonitoring over hospital or clinic visits for monitoring or follow-up because of its convenience [38,43,45,47]. Enhanced clinical decision making and assessment capabilities for HCPs in low-to-middle income countries were described in three telemonitoring interventions [52,54,55]. For these interventions, HCPs such as traditional birth attendants in Guatemala [54]; community-based HCPs in Nigeria, Mozambique, Pakistan, and India [55]; and community health workers in Dominican Republic [52], who have less formal education and training than traditional medical professions, successfully assessed and referred pregnant patients in remote communities to facility-level care as required. These findings are supported by the WHO guidelines for antenatal care, which provide a context-specific recommendation for antenatal home visits to enhance the use of antenatal care services and perinatal health outcomes, especially for those in remote locations with limited access to health services [23]. Evaluations of antenatal health services in Nigeria, Mozambique, India, and Dominican Republic described the need to eliminate possible barriers to access, such as the following: (1) transportation and cost for patients to seek antenatal care [71], (2) reducing delays in referrals from primary health care to more comprehensive antenatal health services [72] by improving the capacity of primary health care facilities to do so [73], and (3) planning for accessible and equitable antenatal health programs [74]. An exploratory study in Pakistan and the WHO guidelines propose the shifting of some antenatal care tasks to other HCPs, such as trained lay health workers (eg, Lady Health Workers in Pakistan) [75], auxiliary nurses, and midwives [23]. Designing more culturally appropriate antenatal programs and government policies may reduce the health risks associated among indigenous pregnant women in countries such as Guatemala, where a significant proportion of the indigenous population resides [76].

### Research Gaps and Suggestions for Future Research

Future studies on the effectiveness of telemonitoring for patients at high risk for HDP need to include more rigorous study designs with larger sample sizes. In addition, longer intervention duration periods may allow for more robust study results and reflect implementation processes more consistent with real-world practice. Telemonitoring interventions for this high-risk group have the potential to enhance antenatal care for women in high-, middle-, and low-income countries. However, addressing implementation and adoption barriers, such as mHealth infrastructure and costs, is key. In addition, which patient group or groups within the high-risk HDP population would benefit most from telemonitoring needs to be determined. More qualitative studies may help to identify this group and guide the researchers in their development and implementation of a telemonitoring intervention.

This review revealed the need for research to establish more robust evidence for the safety and effectiveness of these interventions for this high-risk group. This review proposes 4 recommendations for future research: (1) the implementation of large prospective studies to establish the safety and effectiveness of telemonitoring interventions; (2) additional research to determine the context-specific requirements and patient suitability to enhance patient accessibility to health care services for remote regions and underserved populations; (3) the inclusion of key considerations such as privacy and security in the development and implementation of telemonitoring interventions to better comply with health care information regulations and guidelines; and (4) the implementation of evaluation studies to better understand the effective components of telemonitoring interventions.

### Study Limitations and Strengths

This scoping review has some limitations. It evaluated peer-reviewed journal articles written in English from eight relevant databases, therefore, telemonitoring interventions described in other languages may have been missed. Furthermore, additional search results may have been found in other databases and sources (eg, grey literature, conference proceedings, and books) not included in this review.

Strengths of this review include the use of an experienced information specialist for the development of the search strategy, duplication for each phase of the review (article screening and selection, data extraction, and full-text analysis), and the absence of limitations with respect to year of publication.

### Conclusions

The short- and long-term impacts of HDP on maternal and fetal health are significant and can include multiorgan disease and mortality. Although there are increasingly more studies being published on the telemonitoring of patients at high risk for HDP, this review found only 16 unique interventions on the subject. The current knowledge base of telemonitoring interventions shows some promise for the use of telemonitoring in detecting and managing HDP in patients during and after pregnancy. Specifically, studies indicate that telemonitoring interventions can be feasible, convenient, and cost-effective. However, there is currently very limited evidence on the benefits of telemonitoring on health outcomes. This lack of evidence, combined with mHealth infrastructure and financial barriers, may impede the adoption of these potentially beneficial technologies for patients.

## Appendix

Multimedia Appendix 1 Medical Literature Analysis and Retrieval System Online search strategy.

Multimedia Appendix 2 Overview of included studies on the telemonitoring of women at high risk for hypertensive disorders of pregnancy.

Multimedia Appendix 3 Characteristics and design of the telemonitoring interventions for patients at high risk for hypertensive disorders of pregnancy.

Multimedia Appendix 4 Outcomes of the telemonitoring interventions for patients at high risk for hypertensive disorders of pregnancy.

## Abbreviations

CIHR: Canadian Institutes of Health Research
eHealth: electronic health
HCP: health care provider
HDP: hypertensive disorders of pregnancy
HELLP: hemolysis, elevated liver enzymes, and low platelet
MEDLINE: Medical Literature Analysis and Retrieval System Online
mHealth: mobile health
PRISMA-ScR: Preferred Reporting Items for Systematic Reviews and Meta-Analyses Extension for Scoping Reviews
RCT: randomized controlled trial
WHO: World Health Organization
