# Supplementary material for: Exploring the Use of Telemonitoring for Patients at High Risk for Hypertensive Disorders of Pregnancy in the Antepartum and Postpartum Periods: Scoping Review
Source: JMIR Mhealth Uhealth. 2020 Apr 17;8(4):e15095. doi: 10.2196/15095 (PMC7195666; doi:10.2196/15095)
Supplement: Multimedia Appendix 1 [file mhealth_v8i4e15095_app1.docx]

**Multimedia Appendix 1.** Medical Literature Analysis and Retrieval System Online search strategy conducted on July 30, 2018.

1 Pregnant Women/

2 Pregnancy/

3 exp Pregnancy Complications/

4 Obstetrics/

5 Prenatal Care/

6 peripartum period/

7 postpartum period/

8 (pregnan* or prenatal or pre-natal or perinatal or postpartum or post-partum or puerperium or peripartum* or antepartum or antenatal or postnatal).tw,kw.

9 or/1-8

10 exp Hypertension/

11 exp Blood Pressure Determination/

12 hypertensi*.tw,kw.

13 blood pressure.tw,kw.

14 (preeclampsia or pre-eclampsia or pre eclampsia or gestosis or toxemia*).tw,kw.

15 (pregnan* adj3 ("at risk" or "at-risk" or "high risk" or "high-risk")).tw,kw.

16 or/10-15

17 9 and 16

18 exp Telecommunications/

19 Monitoring, Ambulatory/

20 exp Telemetry/

21 Monitoring, Physiologic/

22 exp Computer Communication Networks/

23 Mobile Applications/

24 Smartphone/

25 (tele-monitor* or telemonitor* or telemed* or tele-med* or teleinterpret* or tele-interpret* or telecomm* or tele-comm* or telemetry).tw,kw.

26 (mhealth* or m-health* or ehealth* or e-health* or telehealth* or tele-health*).tw,kw.

27 (mobile adj3 (health* or technolog* or app* or solution* or phone* or communicat*)).tw,kw.

28 (remote* adj3 (transmi* or transfer* or tele* or monitor* or consult* or follow-up or program* or connect* or web-base* or "web base*" or term)).tw,kw.

29 (monitor* adj3 (home or remote or distan* or ambulatory or tele* or online or on-line or "on line" or phone or digital* or Skype or electronic* or implant* or wireless* or web-base* or "web base*")).tw,kw.

30 (interven* adj3 (remote* or distan* or tele* or online or on-line or "on line" or phone* or digital* or Skype or electronic* or wireless*)).tw,kw.

31 (smartphone* or "smart phone*" or bluetooth* or Internet* or phone* or text messag*).tw,kw.

32 ((app or apps or application*) adj3 (mobile or electronic or software)).tw,kw.

33 ((digital* or electronic* or online* or on-line* or "on line" or Internet) adj3 (health* or solution* or transmit* or transmiss* or transfer* or device* or connect*)).tw,kw.

34 (broadband adj3 (device* or capab*)).tw,kw.

35 (multi-media* or multimedia*).tw,kw.

36 (self monitor* or self-monitor*).tw,kw.

37 or/18-36

38 17 and 37

39 exp animals/ not (exp animals/ and humans/)

40 38 not 39

41 remove duplicates from 40
