# Supplementary material for: Exploring the Use of Telemonitoring for Patients at High Risk for Hypertensive Disorders of Pregnancy in the Antepartum and Postpartum Periods: Scoping Review
Source: JMIR Mhealth Uhealth. 2020 Apr 17;8(4):e15095. doi: 10.2196/15095 (PMC7195666; doi:10.2196/15095)
Supplement: Multimedia Appendix 2 [file mhealth_v8i4e15095_app2.docx]

**Multimedia Appendix 2.** Overview of included studies on the telemonitoring of women at high-risk for HDP

| Reference | Type of Study | Country of Study | N | Population |
| --- | --- | --- | --- | --- |
| Lanssens et al. [48] | Observational | Belgium | Grouped with [49] | Antepartum |
| Ganapathy et al. [43] | Observational | United Kingdom | 50 | Antepartum |
| Perry et al. [36] | Observational | United Kingdom | 166 | Antepartum |
| Tucker et al. [37] | Observational | United Kingdom | 201 | Antepartum |
| Lanssens et al. [49] | Observational | Belgium | 320 | Antepartum |
| Lanssens et al. [50] | Observational | Belgium | 146 | Antepartum |
| Xydopoulos et al. [39] | Observational | United Kingdom | Grouped with [37] | Antepartum |
| Moninex et al. [53] | RCT | Netherlands | 150 | Antepartum |
| Buysse et al. [51] | Observational | Belgium | 415 | Antepartum |
| Dalton et al. [41] | Observational | United Kingdom | 10 | Antepartum |
| Martinez et al. [54] | Observational | Guatemala | 799 | Antepartum |
| Naef et al. [47] | Observational | United States of America | 7 | Antepartum |
| Dunsmuir et al. [55] | Observational | Nigeria, Mozambique, Pakistan, and India | Projected +30,000 | Antepartum |
| Waugh et al. [42] | Observational | United Kingdom | 1 | Antepartum |
| Bonnell et al. [52] | Observational | Dominican Republic | 52 | Antepartum |
| Rhoads et al. [44] | Observational | United States of America | 48 | Postpartum |
| Hinton et al. [38] | Qualitative | United Kingdom | 15 | Postpartum |
| Cairns et al. [40] | RCT | United Kingdom | 91 | Postpartum |
| Hirshberg et al. [45] | Observational | United States of America | 32 | Postpartum |
| Hirshberg et al. [46] | RCT | United States of America | 206 | Postpartum |
