# Supplementary material for: Exploring the Use of Telemonitoring for Patients at High Risk for Hypertensive Disorders of Pregnancy in the Antepartum and Postpartum Periods: Scoping Review
Source: JMIR Mhealth Uhealth. 2020 Apr 17;8(4):e15095. doi: 10.2196/15095 (PMC7195666; doi:10.2196/15095)
Supplement: Multimedia Appendix 3 [file mhealth_v8i4e15095_app3.docx]

**Multimedia Appendix 3.** Characteristics and design of telemonitoring interventions for patients at high-risk for HDP

| Reference | Blood pressure monitor/Validated | Telemonitoring schedule | Technological component(s) | User training |
| --- | --- | --- | --- | --- |
| Lanssens et al. [48] | Withings Wireless BP monitor, pulse O2, Smart Body Analyzer/Not validated | BP and HR (2x/day),  weight (1x/day), activity tracker until delivery | Web-based dashboard | Not specified |
| Ganapathy et al. [43] | Bluetooth enabled BP measurement monitor/Model unspecified | Weekly or more frequently when needed | Android-based mobile application, spreadsheet database on hospital computer | Not specified |
| Perry et al. [36] | Microlife WatchBP home blood pressure machine/Validated | BP and urine. Schedule varied per patient | Hampton Medical smartphone application | Yes |
| Tucker et al. [37] | Microlife WatchBP home blood pressure machine/Validated | BP (2x/day MWF) | NHS Florence telemonitoring service | Yes |
| Lanssens et al. [49] | Withings Wireless BP monitor, pulse O2, Smart Body Analyzer/Not validated | BP and HR (2x/day),  weight (1x/day), activity tracker until delivery | Web-based dashboard | Not specified |
| Lanssens et al. [50] | Withings Wireless BP monitor, pulse O2, Smart Body Analyzer/Not validated | BP and HR (2x/day),  weight (1x/day), activity tracker until delivery | Web-based dashboard | Not specified |
| Xydopoulos et al. [39] | Microlife blood pressure machine/Validated | BP, urinalysis, symptoms. Schedule varied per patient | Hampton Medical smartphone application | Yes |
| Moninex et al. [53] | Unspecified blood pressure monitor model | FHR, BP, temperature (1x/day) | Oxford System 8000 (a computerized system for antenatal heart analysis), telephone modem | Not specified |
| Buysse et al. [51] | Unspecified blood pressure monitor model | BP, CTG, temperature, urine albumin, weight. Unspecified schedule | Vodafone mobile phone, web application, Delphi client application | Yes |
| Dalton et al. [41] | Dinamap 1846 automated blood pressure machine/Model unspecified | BP. HCP-dependent but never more than 4x/day | Motorola 6809-based single board computer, video display unit, telephone modem | Yes |
| Martinez et al. [54] | Omron M7 BP cuff/Validated | BP, HR, O2 saturation, FHR, maternal and perinatal symptoms and clinical signs. Unspecified schedule | Samsung S3 mini, Android mobile application, Onyx II (Model 9560) pulse oximeter, hand-held one-dimensional AngelSounds Fetal Doppler | Yes |
| Naef et al. [47] | Vasoplex home BP monitor/No information found | BP and HR (4x/day) | Patients called BP and HR results to toll-free number, data stored on computer | Yes |
| Dunsmuir et al. [55] | Unspecified blood pressure monitor model | Mean BP, SpO2, gestational age, proteinuria, symptoms. Unspecified schedule | Smartphone, mobile health application, clinical decision support system, server | Yes |
| Waugh et al. [42] | Omron RX automated wrist-mounted BP monitor/Model unspecified | BP. Unspecified schedule | None | Yes |
| Bonnell et al. [52] | Unspecified blood pressure monitor model | BP, weight, urinalysis for glucose and protein, blood glucose, kick count, maternal physical and psychosocial signs and symptoms. Schedule varied per patient | Mobile phone application | Yes |
| Rhoads et al. [44] | Ideal Life Equipment blood pressure monitor, weight scale, pulse oximeter/No information found | BP and HR (2x day), weight and symptoms (1x/day) | Cloud-based portal | Yes |
| Hinton et al. [38] | Microlife WatchBP home blood pressure machine/Validated | BP (2x/day MWF) | NHS Florence telemonitoring service | Yes |
| Cairns et al. [40] | Microlife WatchBP Home, mobile or smartphone/Validated | Daily BP while on medication, then 5 days after treatment cessation, then weekly BP | Smartphone application, study server | Not specified |
| Hirshberg et al. [45] | CVS Pharmacy automated BP cuff or Omron 3 series BP cuff/Not validated | BP (2x/day) for 7 days following discharge | Patients’ mobile phones with unlimited texting | Yes |
| Hirshberg et al. [46] | Omron BP cuff/ Model unspecified | BP (2x/day) for 2 weeks postpartum | Way to Health web-based platform, mobile phone | Yes |

^BP= blood pressure (maternal); HR = Heart rate; MWF = Monday, Wednesday, Friday; NHS = National Health Service; X= times; Validated = Validated for use in pregnancy or preeclampsia; FHR = Fetal heart rate; HCP = Healthcare provider^
