# Supplementary material for: Exploring the Use of Telemonitoring for Patients at High Risk for Hypertensive Disorders of Pregnancy in the Antepartum and Postpartum Periods: Scoping Review
Source: JMIR Mhealth Uhealth. 2020 Apr 17;8(4):e15095. doi: 10.2196/15095 (PMC7195666; doi:10.2196/15095)
Supplement: Multimedia Appendix 4 [file mhealth_v8i4e15095_app4.docx]

**Multimedia Appendix 4.** Outcomes of telemonitoring interventions for patients at high-risk for HDP

| Outcome and reference | Result for patients in telemonitoring group versus control group |
| --- | --- |
| **Maternal and fetal health outcomes (8 studies)** | |
| Lanssens et al. [48] | Spontaneous deliveries were more likely (p=0.001) and inductions were less likely (p=0.006 for univariate and p=0.03 for multivariate analyses) to take place |
| Lanssens et al. [49] | In univariate and multivariate analyses, spontaneous deliveries were more likely (50.00% vs 32.09%, p<0.01) and inductions less likely (32.56% vs 46.51% p<0.01) to take place |
| Perry et al. [36] | No increase in maternal (0.9% vs 3.4%, p=0.245), fetal (25% vs. 24.1%, p=0.902), or neonatal (5.6% vs. 5.2%, p=0.979) adverse outcomes |
| Xydopolous et al. [39] | No difference in adverse maternal, fetal or neonatal outcomes between telemonitoring and control groups but the study was not powered to assess differences in adverse outcomes) |
| Martinez et al. [54] | Rate of reported hypertensive disorder of pregnancy was higher for traditional birth attendants in the early-access group (p=0.03) |
| Moninex et al. [53] | There were more spontaneous deliveries in the domiciliary care group than in the hospital care group (77% vs. 50%, p<0.05) when gestational age was between 37 and 42 weeks |
| Rhoads et al. [44] | No difference in medication adherence between mHealth users and nonusers (p=0.0521) |
| Cairns et al. [40] | Study was not powered to detect differences in safety data, side effects, and quality of life scores |
| **Health system utilization (11 studies)** | |
| Rhoads et al. [44] | mHealth users returned to a medical facility while none of the non-users returned to a medical facility (p=0.0046) |
| Lanssens et al. [48] | Lower number of prenatal admissions until delivery (27.08% vs. 62.24%, p=<0.001) and neonates were less likely to be admitted to the neonatal care intensive care unit (10.42% vs. 27.55% p=0.02) in univariate but not in multivariate analyses |
| Lanssens et al. [49] | Lower number of prenatal admissions until delivery (31.40% vs 57.67%, p<0.01) in univariate and multivariate analyses and fewer prenatal visits (p<0.01) in multivariate analysis |
| Lanssens et al. [50] | Healthcare system costs for remote monitoring were mean €4233.31 (SD €3463.31) per person and mean €4973.69 (SD €5219.00) per person for conventional care (P=0.82), a reduction of €740.38 (14.89%) per person, with savings mainly for the National Institution for Insurance of Disease and Disability (RIZIV) of €848.97 per person (23.18%; mean €2797.42 [SD €2905.18] vs mean €3646.39 [SD €4878.47], P=0.19). |
| Xydopolous et al. [39] | Women using a mobile application to monitor home blood pressure had fewer visits to the day assessment unit (p<0.001) compared to the non-application home blood pressure monitoring and control groups but visited the hypertension clinic more times than the other groups (p<0.001). |
| Moninex et al. [53] | No difference in maternal and neonatal admission rates |
| Buysse et al. [51] | Telemonitoring could replace 14.7 days of in-hospital monitoring and reduce cost by €145,822 per year |
| Hirshberg et al. [45] | No hospital readmissions due to hypertension |
| Martinez et al. [54] | The proportion of successful referral rates to facility-level care were >90% when traditional birth attendants had access to the mHealth platform |
| Perry et al. [36] | Lower number of visits for antenatal services (6.5 vs 8.0, p=0.003) |
| Cairns et al. [40] | 11% (N=5) from the self-management were readmitted to hospital and 7% (N=3) from the control group but study was not powered to detect differences between groups |
| **User experience (5 studies)** | |
| Ganapathy et al. [43] | 90% of patients stated that the self-monitoring technology was easy to use and 78% indicated their preference for self-monitoring compared with conventional hospital or home visits |
| Hinton et al. [38] | Self-monitoring blood pressure empowered and reassured patients, particularly those with a previous history of preeclampsia. Patients were able to incorporate self-monitoring blood pressure into their routines but found it to be a more challenging task in the postpartum. |
| Hirshberg et al. [45] | Patients found that texting was a convenient way for communicating with their obstetrician. Preferred home blood pressure testing and text messaging the results over clinic visits |
| Naef et al. [47] | Home blood pressure monitoring was particularly helpful to patients who lived far (> 60 miles) from the clinic and to those who needed adjustments to blood pressure medications |
| Bonnell et al. [52] | Patients indicated that home visits by the community health worker were comparable to prenatal visits to local health care facilities |
| **Intervention Feasibility (10 studies)** | |
| Rhoads et al. [44] | Higher levels of perceived benefits (p=0.0072) and lower levels of perceived barriers (p=0.0055). There was no difference (p=0.4302) between mHealth users and nonusers in terms of technological anxiety. |
| Ganapathy et al. [43] | Self-monitoring technology presented accurate data and visual cues during the transfer of patient data |
| Tucker et al. [37] | Of the 201 participants who were recruited, 161 (80%) remained in the study at 36 weeks or until delivery. 148 (74%) continued to self-monitor at 20 weeks and 107 (66%) at 36 weeks. Home blood pressure readings were similarly matched to clinic blood pressure readings. |
| Cairns et al. [40] | More likely to reach target blood pressure measurements at 6 weeks. Recruitment rate was 49% (91/186 possible participants). 90% (82/91) of participants completed follow-up visits. Median compliance of submitting daily blood pressure readings was 85%. Accuracy of submitted daily readings was 87%. |
| Dalton et al. [41] | 90 blood pressure measurements were transmitted from patients’ homes. On average, home blood pressure values were lower than clinic blood pressure. |
| Hirshberg et al. [45] | 84% (27/32) participants texted at least one blood pressure measurement. Patients more likely to text blood pressure values in the first few days of monitoring than the last few days |
| Hirshberg et al. [46] | 10 days postpartum, 92.2% from the texting group sent one blood pressure measurement compared to 43.7% from the control group. 84% of the texting group met American Congress of Obstetricians and Gynecologists (ACOG) criteria for blood pressure monitoring |
| Naef et al. [47] | Home blood pressure values correlated with clinic values |
| Waugh et al. [42] | Home blood pressure monitoring allowed for accurate and frequent blood pressure measurements |
| Bonnell et al. [52] | Consistent internet connection was an issue in remote areas |
